# Influence of Biological Factors on Connectivity Patterns for *Concholepas concholepas* (loco) in Chile

**DOI:** 10.1371/journal.pone.0146418

**Published:** 2016-01-11

**Authors:** Lysel Garavelli, François Colas, Philippe Verley, David Michael Kaplan, Beatriz Yannicelli, Christophe Lett

**Affiliations:** 1 Institut de Recherche pour le Développement (IRD), UMI 209 UPMC UMMISCO, Centre de Recherche Halieutique Méditerranéenne et Tropicale, Sète, France; 2 Institut de Recherche pour le Développement (IRD), LOCEAN-IPSL, UPMC, Paris, France; 3 Institut de Recherche pour le Développement (IRD), UMR MARBEC, Centre de Recherche Halieutique Méditerranéenne et Tropicale, Sète, France; 4 Virginia Institute of Marine Science, College of William & Mary, Gloucester Point, Virginia, United States of America; 5 Centro de Estudios Avanzados en Zonas Aridas (CEAZA), Facultad de Ciencias del Mar, Universidad Católica del Norte, Coquimbo, Chile; 6 Centro Universitario Región Este, Universidad de la República, Montevideo, Uruguay; National Cheng-Kung University, TAIWAN

## Abstract

In marine benthic ecosystems, larval connectivity is a major process influencing the maintenance and distribution of invertebrate populations. Larval connectivity is a complex process to study as it is determined by several interacting factors. Here we use an individual-based, biophysical model, to disentangle the effects of such factors, namely larval vertical migration, larval growth, larval mortality, adults fecundity, and habitat availability, for the marine gastropod *Concholepas concholepas* (loco) in Chile. Lower transport success and higher dispersal distances are observed including larval vertical migration in the model. We find an overall decrease in larval transport success to settlement areas from northern to southern Chile. This spatial gradient results from the combination of current direction and intensity, seawater temperature, and available habitat. From our simulated connectivity patterns we then identify subpopulations of loco along the Chilean coast, which could serve as a basis for spatial management of this resource in the future.

## Introduction

In coastal waters, benthic invertebrate populations are spatially distributed in a patchy habitat. Local persistence and spatial distribution of such populations are largely influenced by the capacity and scales of exchange of individuals between patches, *i*.*e*. the connectivity [1]. Most marine invertebrate benthic species are characterized by a dispersive pelagic larval phase and a relatively sessile benthic adult phase. As such, larval dispersal is essential to population maintenance in these species. Connectivity through larval dispersal (hereafter referred to as larval connectivity) depends on both physical transport and biological factors, such as larval behavior, larval mortality, larval growth and availability of spawning and recruitment habitats [2]. Due to the inherent complexity of conducting field and experimental observations, biophysical individual-based models have been developed to investigate the importance of different biotic and abiotic processes for larval connectivity ([3–6]). Here, we use such a model to study the separate and cumulative effects of important larval and adult biological traits on larval connectivity for the marine gastropod *Concholepas concholepas* (commonly called loco), a keystone species of rocky intertidal food webs highly exploited in Chile [7,8].

Larval behavior has been most frequently included in biophysical models in the form of vertical migration [9,10]. Indeed, larvae of many species are able to respond to physical (salinity, temperature) or biological (food availability, predator evasion) conditions by migrating in the water column. Generally, including a diel vertical migration (DVM) scheme in a larval dispersal model for coastal benthic species increases larval settlement and decreases offshore losses (in Chile [11]; in the Irish Sea [12]). In the case of organisms that inhabit coastal upwelling areas, it is often thought that improved settlement success with DVM is linked to larvae being located below the Ekman layer, thereby avoiding offshore transport or even being transported onshore. Nevertheless, the robustness of increased settlement due to DVM has rarely been tested in a comparative framework including other mechanisms affecting larval transport and different hypotheses regarding the depth range of DVM [13,14].

Another biological factor that largely influences recruitment is larval survival. Natural mortality of larvae is believed to be high, between 1.6 and 35.7% d^-1^ for marine invertebrate larvae [15]. In a study focusing on several marine invertebrate species between the North Sea and the Baltic Sea, the percentage of larval mortality was evaluated around 90% over the larval dispersal phase [16]. Including a daily mortality rate in biophysical models for benthic species can impact simulated larval distributions [17] and induce equivalent larval losses as do hydrodynamic processes [18]. It is therefore important not to ignore the potential consequences of larval survival on spatial patterns of larval connectivity.

Finally, larval growth is thought to be one of the main factors affecting the planktonic larval duration (PLD) and ultimately larval survival and recruitment. Water temperature has in turn been demonstrated to have a strong relationship with larval growth in a wide variety of species [19,20,6]. Therefore, temperature is often used as a proxy for larval growth rates in biophysical models [21–23].

To our knowledge, few comparative studies exist examining the relative impacts of these diverse biological factors on connectivity of marine benthic invertebrates. We therefore propose to use a biophysical model integrating these factors to study dispersal and connectivity of loco. Until the end of the 80’s, loco’s fishery was one of the most valuable benthic fisheries in Chile [24]. During the 1960’s, loco fishery typically supplied local markets, with annual landings around 5,000 t (Sernapesca, National Service of Agriculture and Fisheries in Chile, 2012). In 1976, the international market started to be interested in this resource and landings increased steeply reaching a maximum of 25,000 t in 1980. Between 1983 and 1988, fishing effort continued to increase whereas total landings started to decline. The resource was recognized as overexploited and the tremendous decrease in catch per unit effort led to a complete fishery closure during four years between 1989 and 1992 [25]. After re-opening the fishery in 1993, loco landings never recovered the previous levels of extraction despite stringent management regulations. Different management plans have been applied since then to ensure the sustainability of the fishery, but no attempt has been made to explicitly link strategic management scales with relevant population scales. The current management plan focuses on the establishment of spatially-explicit, locally-managed zones referred to as “Management and Exploitation Areas for Benthic Resources” (MEABR) whose spatial extent is within 10 km [26].

The biology of loco larval stages is poorly known, nevertheless DVM patterns have been established at early [27] and late [28] larval stages of loco, and latitudinal differences in PLD and fecundity have been reported. The loco biogeographic range spreads for over 40 degrees of latitude, where it successfully develops under very different temperature regimes [29]. Surface temperatures range from 13°C to 20°C from 32°S to 18°S, and from 11°C to 13°C from 56°S to 32°S [29]. This latitudinal temperature gradient is consistent with field and laboratory estimates of a shorter (2 to 4 months) larval dispersal phase in south-central Chile and a longer one (12 months) further south in the fjords [30–32]. For loco, the end of larval dispersal, *i*.*e*. settlement, occurs when competent larvae metamorphose [30]. Therefore, loco larval settlement occurs within a restricted final size range. A previous biophysical modeling study developed for this species [33] has suggested that loco connectivity is affected by larval release depth and PLD when larvae were passively transported and PLD values were homogeneous throughout the coast. However, as previously described, loco dispersal is driving by complex processes such as DVM and a long, temperature-dependent larval dispersal phase. Larval behavior, growth, and mortality are all expected to have significant effects on loco connectivity patterns and may therefore influence loco population dynamics. In addition, a latitudinal gradient in loco adult fecundity has been observed with lower fecundity in the north than in the south of Chile [34]. It has been hypothesized that this gradient compensates partly for temperature- and transport-driven differences in larval mortality along the Chilean coast [33]. To disentangle the effects of all the biological processes mentioned above, we compare simulated larval dispersal and connectivity patterns for loco with and without each of these.

## Material and Methods

### Hydrodynamic model

We used the Regional Oceanic Modeling System (ROMS) in its “UCLA” version [35,36] to simulate the oceanic circulation of the Peru-Chile Current System. ROMS is a free-surface split-explicit model that solves the hydrostatic primitive equations and uses terrain-following curvilinear vertical coordinates. We have applied a quasi-equilibrium approach using monthly climatological means for both atmospheric forcing and open-ocean model boundaries. Previous studies have successfully used this approach to simulate the mean circulation and the mesoscale dynamics of eastern boundary upwelling systems (*e*.*g*. [37–40]). Here, the model is run over a large domain covering the South-East Pacific (from 15°N to 41°S and from 100°W to the South American coast) with a horizontal resolution of 7.5 km. Monthly-mean surface forcing was taken from the COADS climatology for heat and freshwater fluxes [41], and the QSCAT scatterometer-based climatology SCOW for wind stress [42]. Open-boundary forcing was a monthly climatology taken from SODA [43] over the period 2000−2006. The model was integrated over a period of 13 years. The first 3 years are considered as the spin-up phase and hence are not included in the analysis. Time series of current velocities and temperature were averaged and stored every 3 days. We refer to Colas *et al*. [44,45] for more details on these simulations and their evaluation against observations.

### Biophysical model

**Fig 1:**
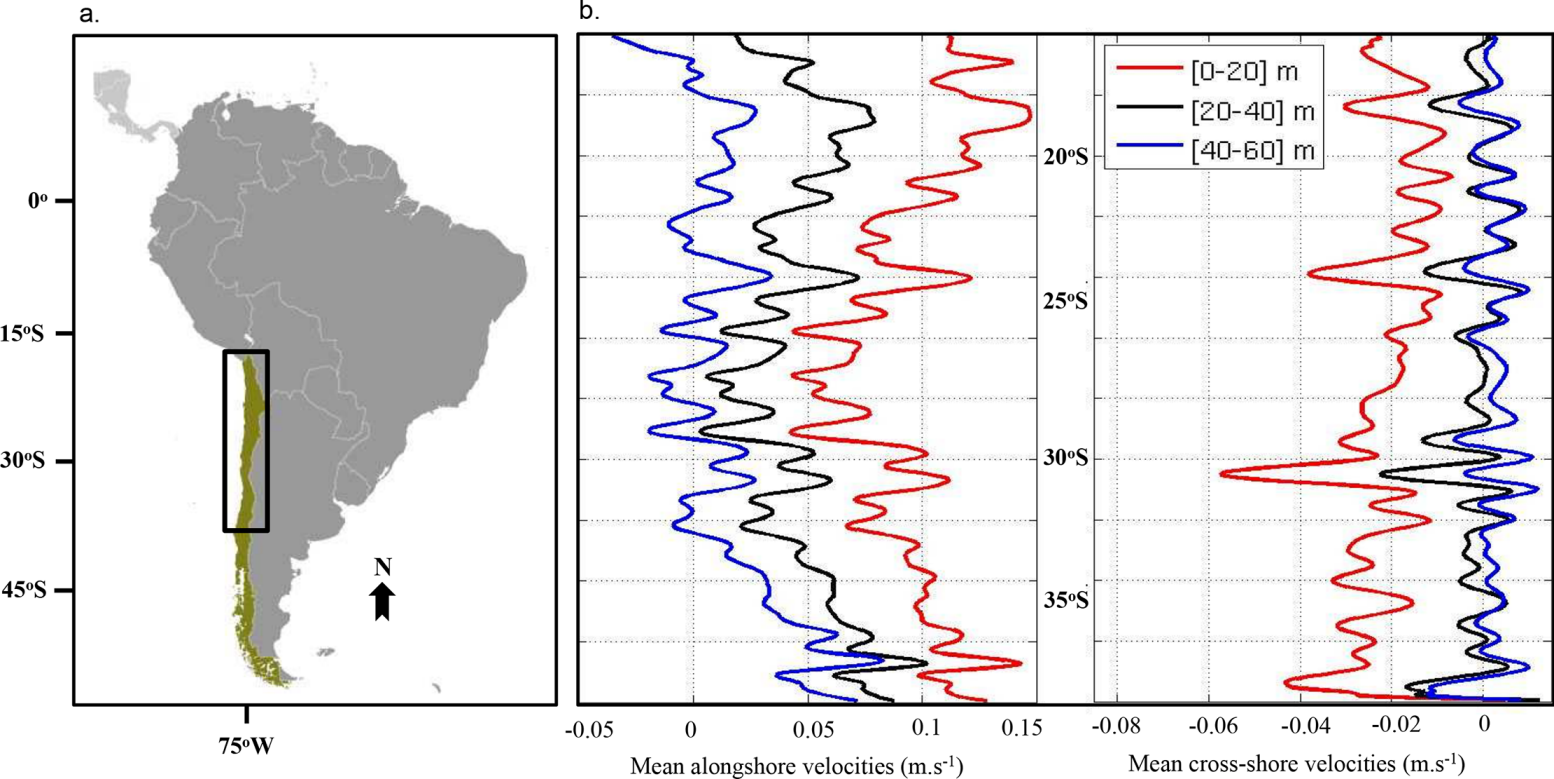
(a.) Map of South America with Chile in green. The study domain is delimited by a black box, from 16°S to 38°S. (b.) On the left: Mean annual velocities of alongshore currents between 16°S and 38°S derived from the hydrodynamic model (averaged over the settlement area width) for depth range 0–20 m (red line), 20–40 m (black line), 40–60 m (blue line). On the right: Mean annual velocities of cross-shore currents between 16°S and 38°S derived from the hydrodynamic model (averaged over the settlement area width) for depth range 0–20 m (red line), 20–40 m (black line), 40–60 m (blue line).

Modelling loco larval dispersal was performed using the individual-based offline Lagrangian tool Ichthyop v. 3.1 [46]. Using a forward-Euler advection scheme with velocity fields derived from ROMS, locations of each virtual larva (latitude, longitude and depth) were tracked every hour in three dimensions along the Chilean coast (Fig 1A). Horizontal diffusion was added following Peliz *et al*. [9] to represent diffusion on spatio-temporal scales below the model resolution (with a turbulent dissipation rate є = 10^−9^ m^2^.s^-3^). Release areas were designed as sections of 0.25° of latitude all along the Chilean coast from 16°S to 38°S stretching from the coast to the 500 m isobaths, a compromise between knowledge of loco spawning areas and the spatial resolution of bathymetry in the oceanographic model. Over these areas, 100,000 individuals were randomly released each month from January to December during 4 years that were randomly selected among years 4−13 of ROMS. For model configurations without growth included, the criterion used for settlement was for larvae to be in settlement areas (chosen as being the same as release areas) anytime in the last 20 days of a fixed PLD. For model configurations with growth included, the criterion was for larvae to be in settlement areas and to be larger than the minimal size for settlement (see below). Simulation results are represented as connectivity matrices. Values of the connectivity matrix C_*ij*_ were calculated as the percentage of virtual larvae released from area *j* that are transported to area *i*. For all model configurations, a relative larval transport success was calculated for each release area: the number of larvae successfully transported to a destination area was summed and normalized by the maximum value of transport success among all release areas for that connectivity matrix. We also calculated mean dispersal distances of settled larvae in relation to their release areas. Dispersal distance was calculated as the difference in kilometers between the location of settlement (latitude and longitude) and the location of release for each larva. We finally compared our results with the results obtained by Garavelli *et al*. [33] using a model with passive transport. We refer to their results when larval spawning depth was set between 0 and 20 m as configuration M0.

### Model configurations

In our study, six model configurations were evaluated, referred to as M1–M6 (Table 1).

**Table 1:** Summary of processes and parameters used in the different model configurations. DVM = diel vertical migration.

Model configuration	Release and destination areas	Release depth	Biological process implemented	Dispersal duration	Criteria for settlement
M0 (From Garavelli et al. [31])	From 15.85°S to 31.10°S One area = 0.25o of latitude	0–20 m	None	80 days	Last 20 days
M1	As M0	As M0	DVM 0–20 m (a), 0–40 m (b), 0–60 m (c) From the first day of simulation	80 days and 140 days	Last 20 days
M2	As M0	As M0	Linear larval growth function depending on temperature	140 days	Larval size (1900 **μm)**
M3	As M0	As M0	As M2 + habitat limitation	As M2	As M2
M4	As M0	As M0	As M3 + relative fecundity	As M2	As M2
M5	As M0	As M0	As M4 + DVM 0–20 m From the first day of simulation	As M2	As M2
M6	As M0	As M0	As M5 + larval mortality	As M2	As M2

Model configuration M1 was designed to study the effect of larval DVM on loco connectivity patterns. Three DVM amplitudes were chosen to encompass the loco larval vertical distribution: 0–20 m (M1a), 0–40 m (M1b), and 0–60 m (M1c). The DVM behavior implemented was applied every day along the whole PLD [27,28]. In M1, the PLD used was 120 days corresponding to the maximum value in our study domain found by Moreno *et al*. [31]. Moreover, we added a competency period. To find a suitable habitat for settlement, loco larvae are able to delay their metamorphosis for hours to weeks [47]. For the gastropod *Crepidula fornicata*, the duration of the competency period was observed to be between 20 to 30 days [19]. Using both references, we chose a competency period of 20 days as a reasonable approximation for loco larvae. In M1, transport success of the larvae in the settlement areas was therefore computed between 120 days and 140 days, subsequently referred as PLD of 140 days. A shorter PLD (80 days) was also tested, like in the previous modeling study [33].

Model configuration M2 aimed at investigating the effect of larval growth in relation to temperature on the connectivity patterns. To develop a growth model for loco larvae we used the relationship established by O’Connor *et al*. [20] between PLD and temperature from a meta-analysis:

$$\text{ln}\left(PLD(T)\right)=\beta -1.34\times \text{ln}\;\left(\frac{T}{15}\right)-0.28\times {\left(\text{ln}\;\left(\frac{T}{15}\right)\right)}^{2}$$ (1)

where T is temperature in °C, β is a species-specific parameter, and PLD is in days.

For loco larvae, under laboratory conditions at T = 16°C, PLD was estimated to be approximately 90 days [30]. Therefore, from Eq (1), we determined that β = 4.587. For the range of temperatures between 11°C and 22°C experienced by loco larvae in the study area [29,48], Eq (1) gives PLD ranging from around 145 days to 56 days.

To transform these PLD values into growth rates (GR in μm day^-1^), we assumed linear growth from size at hatching (250 μm) to size at settlement (1900 μm) based on DiSalvo [30] and Manríquez *et al*. [49]:

GR(T)=(1900−250)/PLD(T) (2)

In the loco larval dispersal model, for every time step Δt = 1/24 day, we calculated GR(T) based on temperature at the virtual larva location obtained from the hydrodynamic model, and then updated the larva length (L in μm) using:

$$L_{t+\Delta t}=L_{t}+GR(T)\times \Delta t$$ (3)

In M2, individuals were released between 0 and 20 m depth and were tracked for 140 days.

With the connectivity matrix resulting from M2, we assessed the additional potential effects on connectivity of spatial heterogeneity along the Chilean coast of loco habitat (M3) and relative fecundity (M4). For loco habitat, we evaluated the proportion of rocky shore within each latitudinal band of 0.25° as explained in Garavelli *et al*. [33]. We then multiplied the connectivity values of each column and each row by the corresponding proportion of available habitat to adjust larval production and larval settlement, respectively, for limited habitat area.

The effect of loco fecundity was estimated using Fernández *et al*.’s [34] study of loco embryo packing in egg capsules. They observed fewer loco embryos per unit area of capsule in the North than in the South of Chile with a break around 29−30°S. They found no difference in the number of loco embryos per unit area of capsule among the sampling sites north of this break, nor among the southern sampling sites. From these results, we assessed the ratio between the mean number of loco embryos per unit area of capsule as 1.68 between the northern and southern regions. From 29.35°S to the southern limit of our study domain, we multiplied the values of each column (representing release areas in the connectivity matrix) by this ratio to adjust our connectivity values for loco relative fecundity.

We then added the effect of DVM on connectivity (M5) by including a larval DVM scheme between 0 and 20 m into the larval dispersal model as described for M1. We chose to show the results obtained with this DVM range only due to similar qualitative patterns observed between this model configuration and those with larger DVM vertical depth ranges.

Finally, to assess the effect of larval mortality on loco larvae connectivity (M6), we used a mortality rate of 0.12 d^-1^ estimated for veliger larvae of *Mytilus edulis* by Rumrill [15], in the absence of a corresponding estimate for veliger loco larvae. The connectivity matrix with mortality was constructed by first weighting each larvae by (1 − 0.12)^*age*^ where age is the age at settlement in days, and then calculating the connectivity matrix as the weighted sum over larvae as a function of release and settlement locations divided by the number of larvae released from the site of origin.

### Identification of subpopulations

We used the methodology of subpopulations identification developed by Jacobi *et al*. [50] to identify approximately independent subpopulations from the connectivity matrices for each model configuration (M1 to M6). The algorithm used calculates a set of contiguous subpopulations that minimize connectivity between subpopulations for a given desired level of aggregation. Jacobi *et al*.’s [50] method uses the mean connectivity between an area *i* and an area *j*, calculated as the average connectivity between these areas $\frac{C_{ij}+C_{ji}}{2}$. In the algorithm, all connectivity matrix columns can be normalized, but this option was not used here as it would delete the effect of habitat limitation. In a limited number of cases the algorithm identified a single, isolated area as a subpopulation; in this situation, we regrouped the area with the closest subpopulation. We refer to Garavelli *et al*. [33] for a more detailed description of the method.

### Statistics

In M1 and M2, we performed a sensitivity analysis to test the effect of release area and release month on the simulated values of settled larvae using a multifactor analysis of variance (ANOVA). In all cases, p values < 0.05 were taken to indicate significant results.

## Results

### Mean velocity currents

Mean alongshore and cross-shore current speeds derived from the hydrodynamic model used in this study are represented in Fig 1B between 16°S and 38°S. Logically, the intensity of the currents decreases with depth ranges. For all depth ranges, current velocities are higher at the southern limit of the domain. Mean positive values indicate that alongshore currents in the depth ranges 0−20 m and 20−40 m are mostly oriented northward along the domain. This is also true between 40 and 60 m depth in the southern limit, but north of ~ 32.5°S currents are frequently southwards. For all depth ranges, lower values of alongshore speed are observed between 25°S and 29°S. Around this minimum, two peaks of higher currents are particularly visible at 24°S and between 29.5°S and 30−31°S. There are also peaks closer to the limits at 19°S and 37°S. Mean-cross-shore velocities are negative along the entire domain for the depth range 0–20 m indicating an offshore orientation of the currents consistent with a surface Ekman flow forced by the upwelling favorable wind. Cross-shore velocities intensities decrease for 20–40 m and 40–60 m depth ranges. In these depths ranges, we can observe an alternation of weak offshore and onshore currents. Three peaks of negative (offshore) values are observed around 24°S, 31°S, and 37°S mainly for the depth ranges 0–20 m and 20–40 m.

### Vertical distribution of settlers

**Fig 2:**
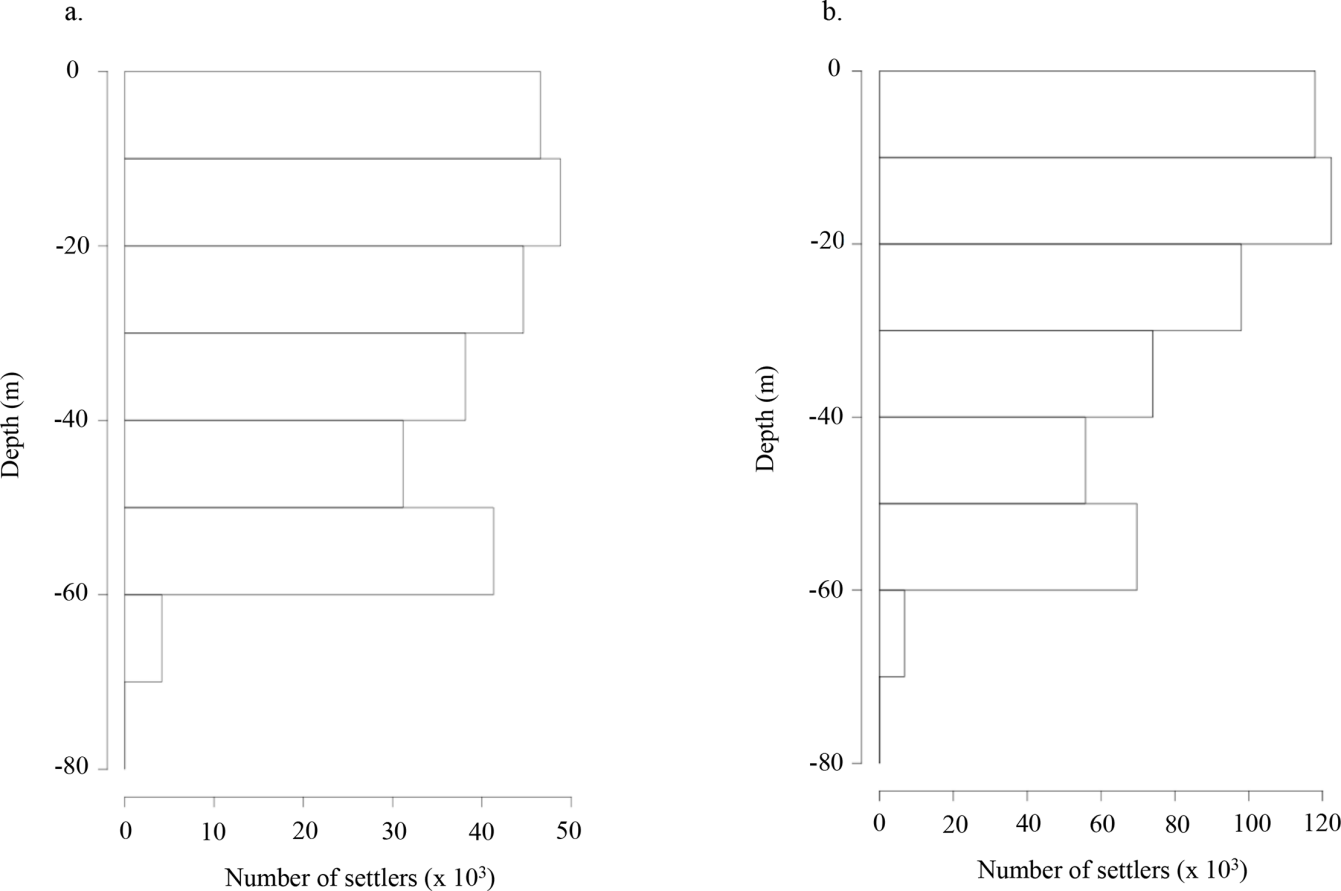
Number of settled larvae per depth for (a.) model configuration M0 (passive transport; figure is obtained using the results from Garavelli *et al*. [33]) and (b) model configuration M2 (with growth).

The vertical distribution of settled larvae in the water column is shown for M0 (*i*.*e*. from Garavelli *et al*. [33]; passive transport) in Fig 2. Settled larvae are homogeneously found between 0 and 60 m depth. The maximum depth of settled larvae is 76.79 m. For M1 a, b, and c, the depth ranges of the settled larvae are constrained by the DVM depth ranges (0–20 m for M1a, 20–40 m for M1b, 40–60 m for M1c). For M2, the number of settlers is higher and their depth distribution is slightly shifted to the upper depth levels with a maximum depth of 76.55 m. A similar pattern is obtained for M3 and M4, whereas for M5 and M6 the depth distribution is again imposed by the DVM pattern.

### Loco larval dispersal distances

**Fig 3:**
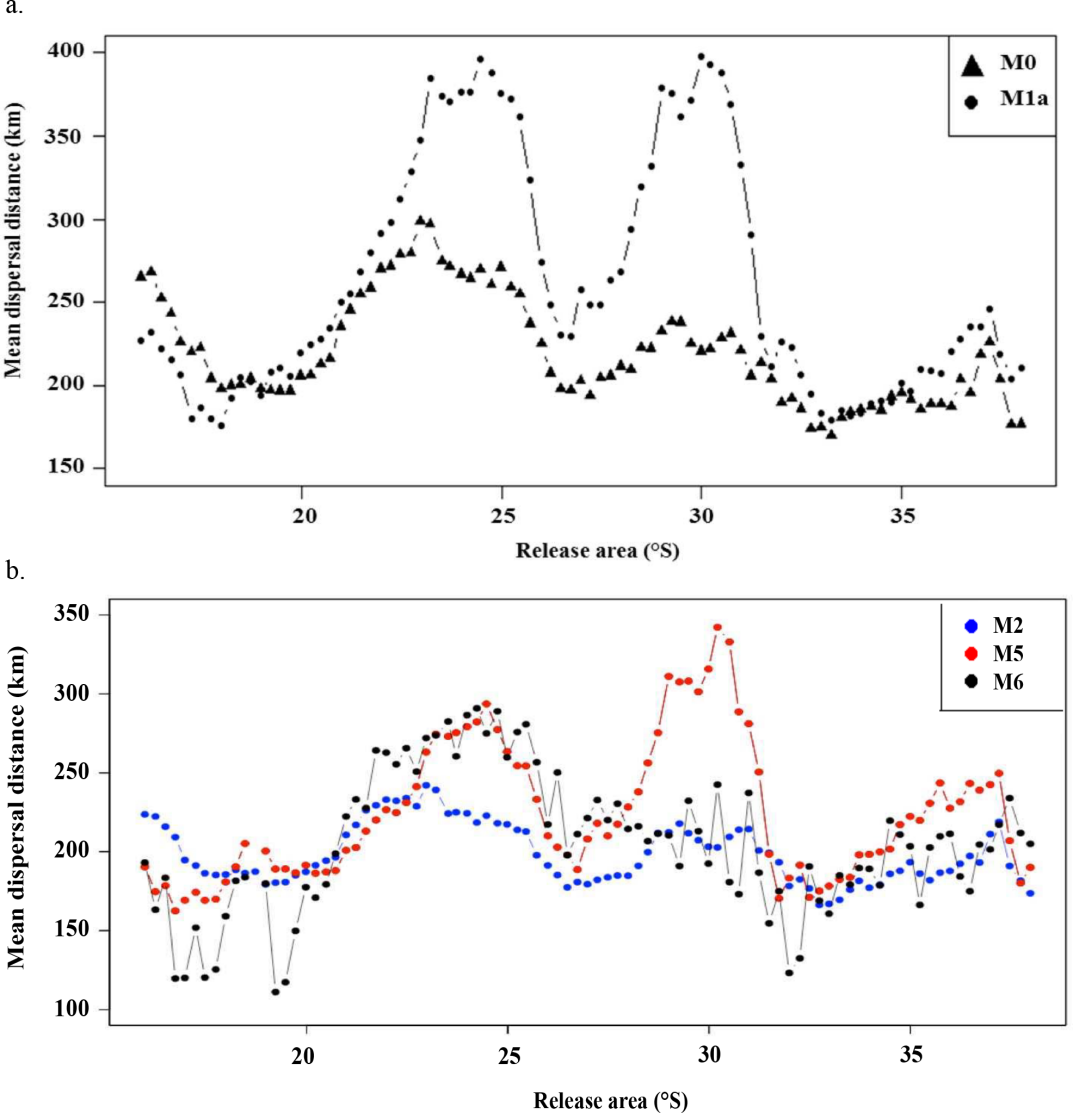
Mean dispersal distances traveled by settled loco larvae in relation to their release areas. (a.) For model configurations M0 (passive transport; figure is obtained using the results from Garavelli *et al*. [33]) and M1a (with DVM 0–20 m). (b.) For model configurations M2 (including growth), M5 (adding habitat, fecundity, and DVM), and M6 (adding mortality).

Results of mean dispersal distances of settled larvae in relation to their release area are shown for M0 (*i*.*e*. from Garavelli *et al*. [33]; passive transport) and M1a (*i*.*e*. including DVM depth range between 0 and 20 m) in Fig 3A. Mean dispersal distances along the study domain are 220 km and 262 km for M0 and M1a, respectively. Dispersal distances are lower for M0 mainly for the release zones located between 23°S and 25°S and between 29°S and 31°S, and slightly higher toward the northern limit of the study domain (Fig 3A). Similar patterns of dispersal are observed for M1b (DVM = 0−40 m; not shown) and M1c (DVM = 0−60 m; not shown), although dispersal distances values are modulated by the DVM depth range (Table 2).

**Table 2:** Summary of results obtained for all model configurations: M0, M1a, M1b, M1c, M2, M3, M4, M5, and M6 (see Table 1 for details).

Model configurations	M0	M1a	M1b	M1c	M2	M3	M4	M5	M6
Mean connectivity values (%)	0.06	0.03	0.04	0.05	0.13	0.09	0.1	0.07	5.69 × 10^−7^
Mean dispersal distance (km)	219.88	262.35	256.27	248.43	198.91	198.77	198.77	224.31	205.38
Maximum dispersal distance (km)	298.46	397.6	364.51	333.74	242.02	249.85	249.85	342.03	288.34
Minimum dispersal distance (km)	170.05	175.99	170.51	184.74	166.29	158.87	158.87	162.23	117.25

Adding larval growth (M2) to the model results in higher mean connectivity values and lower dispersal distances (Table 2) but produces little change on how dispersal distances change with release areas as compared to M0 (Fig 3B). Adding habitat limitation (M3) and relative fecundity (M4) has little effect (figures not shown; Table 2), whereas successively including DVM (M5) and larval mortality (M6) significantly changes both the mean (Table 2) and spatial pattern (Fig 3B) of dispersal distances. Adding DVM notably increases dispersal distances (Table 2), particularly between 22°S and 32°S (Fig 3B) where settled larvae are transported 100 km to 150 km further than in M2. By contrast, dispersal distances for M5 are lower than those for M2 towards the northern limit of the study domain. Maximum dispersal distances are observed for M5 for larvae released around 30°S, but dispersal distances are notably lower in this region when larval mortality is included in the model (M6; Fig 3B).

### Effect of DVM on loco larval settlement

In model configurations M1a, M1b, and M1c, release month is the factor explaining the most variance in the proportion of settled larvae for both values of dispersal duration (Table 3). The most favorable release period for loco settlement success is between January and June (austral summer and autumn; Fig A in S1 File). Release areas with the highest percentage of successful loco larvae are located between 17°S and 27°S and between 32°S and 38°S. Percentage of variance explained by release month increases with increasing DVM range (Table 3). Similar spatio-temporal patterns are observed for M1b and M1c (not shown) although mean connectivity values increase with increasing DVM range (Table 2).

**Table 3:** Model configurations M1a, M1b, M1c, and M2 (see Table 1 for details): sensitivity analysis (ANOVA) on proportion of settled larvae with release area and release month factors.

Factor	Degrees of freedom	Model configuration	Percentage variance explained	p value
Release area	88	M1 a PLD 140 days	24.91	< 2.2 × 10^−16^
Release month	11		30.01	< 2.2 × 10^−16^
Release area × release month	968		17.47	< 2.2 × 10^−16^
Residuals	3204		27.6	
Release area	88	M1b PLD 140 days	19.11	< 2.2 × 10^−16^
Release month	11		38.22	< 2.2 × 10^−16^
Release area × release month	968		16.42	< 2.2 × 10^−16^
Residuals	3204		26.22	
Release area	88	M1c PLD 140 days	15.66	< 2.2 × 10^−16^
Release month	11		46	< 2.2 × 10^−16^
Release area × release month	968		14.59	< 2.2 × 10^−16^
Residuals	3204		23.74	
Release area	88	M2	48.88	< 2.2 × 10^−16^
Release month	11		20.44	< 2.2 × 10^−16^
Release area × release month	968		8.05	6.5 × 10^−4^
Residuals	3204		22.6	

**Fig 4:**
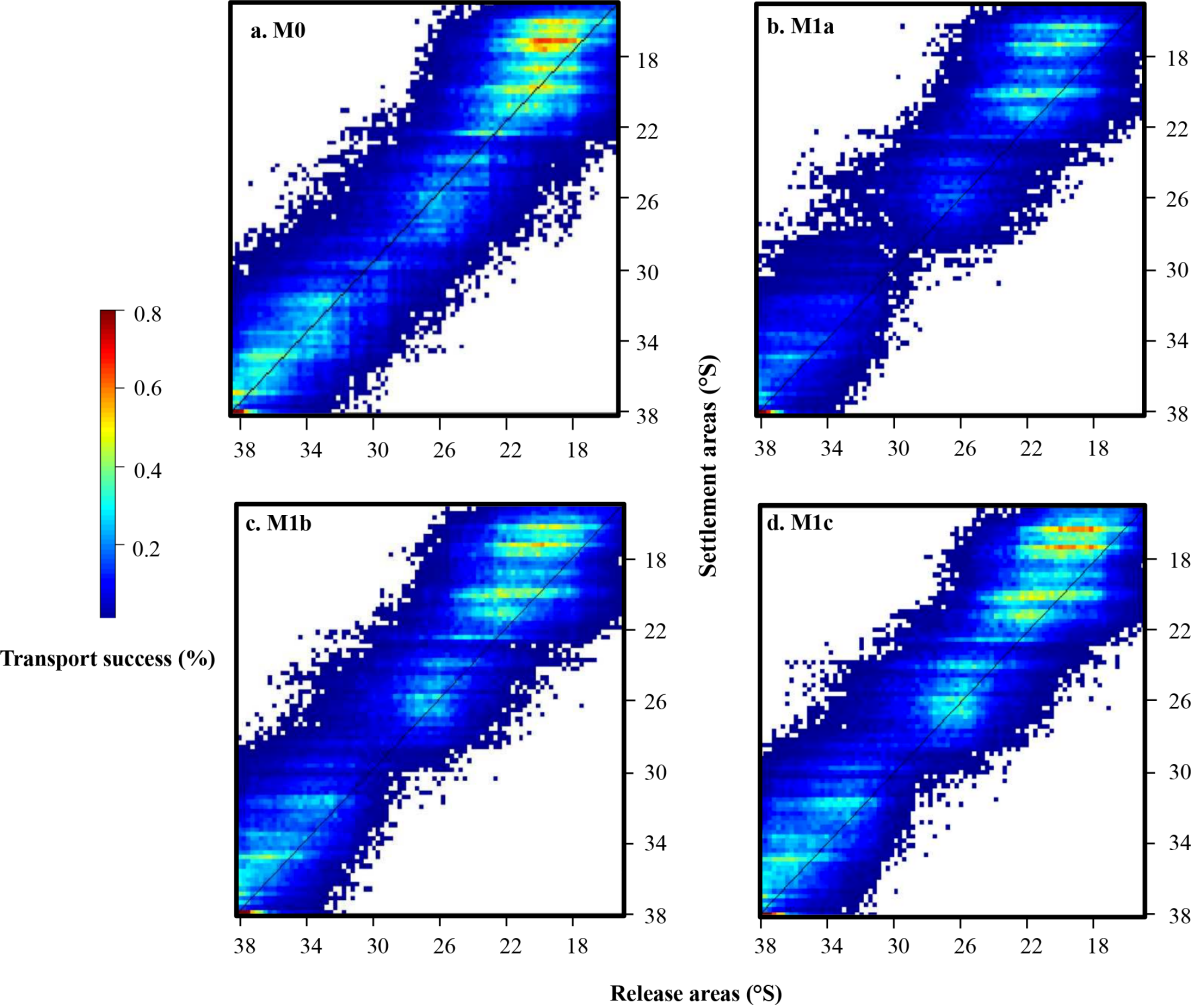
Connectivity matrices obtained for model configurations M1a (with DVM 0–20 m; b.), M1b (with DVM 0–40 m; c.), and M1c (with DVM 0–60 m; d.). (a.) Reprinted from Garavelli *et al*. [33] under a CC BY license, with permission from Elsevier, original copyright 2014. (model configuration M0, passive transport).

Compared to the connectivity matrix obtained with passive transport (M0; Fig 4A), the DVM scheme included in the dispersal model leads to a decrease in connectivity values, especially from 23°S to the southern limit of the domain (M1a; Fig 4B). Three hotspots of released larvae that successfully settle are observed at 16–22°S, 24–27°S, and 32–38°S (M1b, Fig 4C; M1c, Fig 4D). Generally, similar qualitative patterns are observed between model configurations with different DVM depth ranges. For all DVM schemes, connectivity values are lowest in the southern part of the domain and higher values of connectivity are obtained above the diagonal, indicating that virtual larvae are mainly transported to the North. This northwards transport is less predominant when DVM is not included, except in the northernmost region.

### Effect of combined factors on loco larval settlement

When larval growth is integrated into the model (M2), most larvae settle at 1.9 mm, the minimum length set for settlement criterion (Fig B in S1 File). The mean length at settlement is 2.31 mm ± 0.47 mm and the maximum length is 4.8 mm. As opposed to M1, in M2, the factor explaining the most variance in the proportion of settled larvae is release area (Table 3).

**Fig 5:**
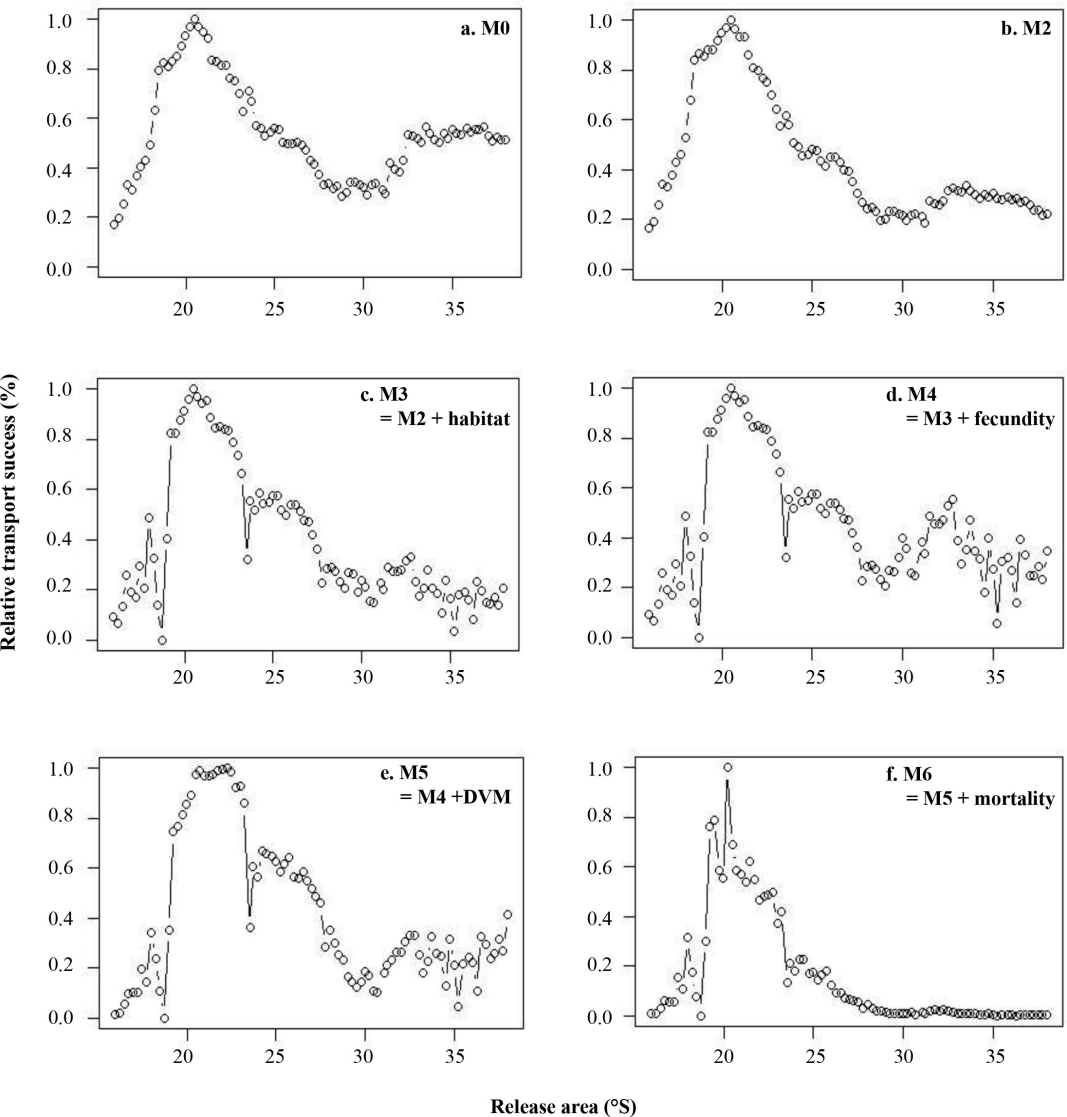
Relative transport success obtained for model configurations M2 (with growth; b.), M3 (adding habitat; c.), M4 (adding fecundity; d.), M5 (adding DVM; e.), and M6 (adding mortality; f.). Panel a. is obtained using the results from the model configuration M0 (passive transport) in Garavelli *et al*. [33]. For each release area, transport success is calculated relative to the maximum value for each connectivity matrix.

**Fig 6:**
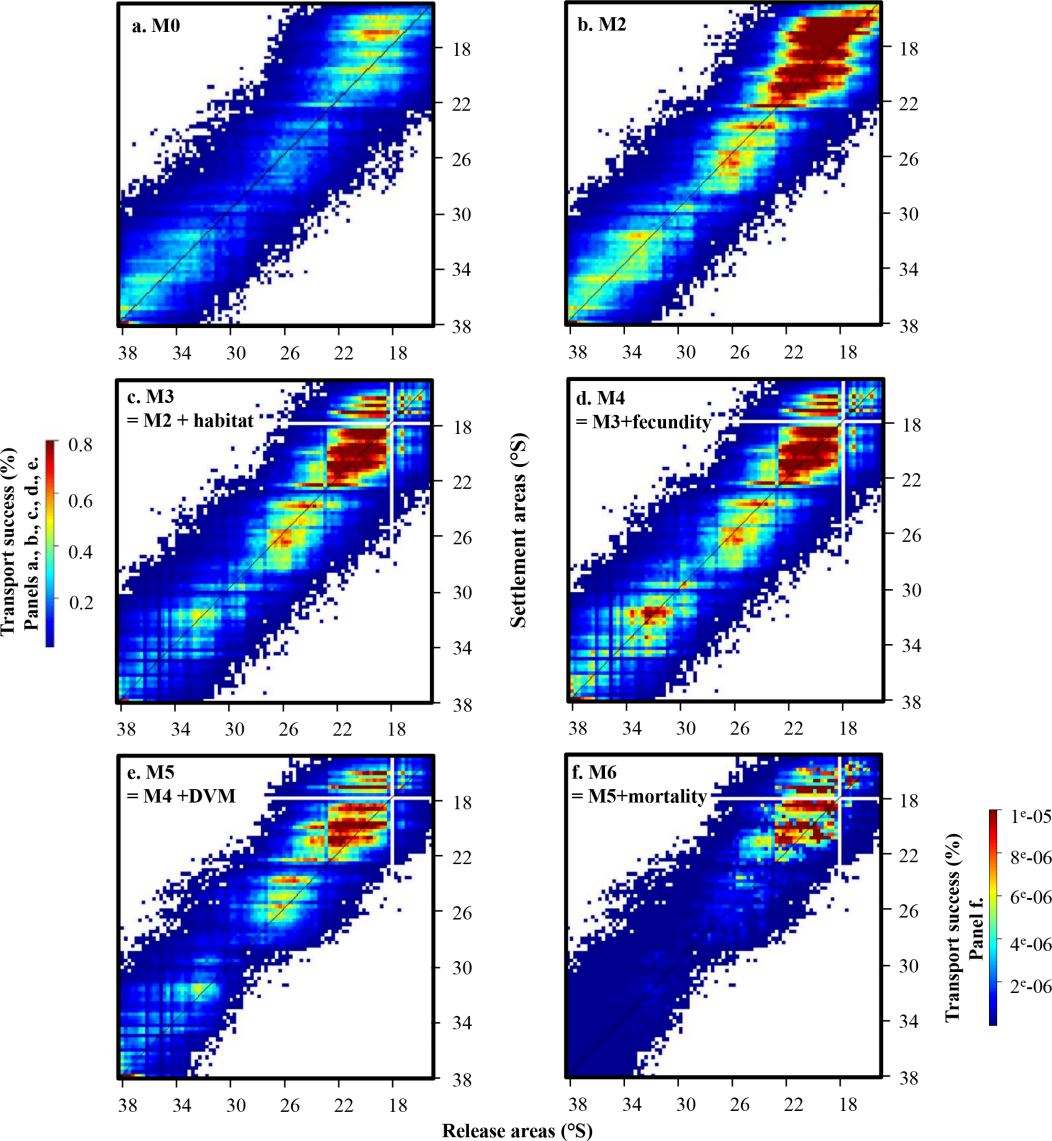
Connectivity matrices obtained for model configurations M2 (with growth; b.), M3 (adding habitat; c.), M4 (adding fecundity; d.), M5 (adding DVM; e.), and M6 (adding mortality; f.). (a.) Reprinted from Garavelli *et al*. [33] under a CC BY license, with permission from Elsevier, original copyright 2014. (model configuration M0, passive transport). Note that the colour bar is different for panel f.

Though all model configurations shared some overall qualitative spatial trends in transport success, a number of significant qualitative and quantitative differences exist between model configurations. For all model configurations, highest values of connectivity are observed in the northern part of the domain, between 19°S and 22°S (Figs 5B–5F and 6B–6F). Spots of higher connectivity are also obtained between 24°S and 27°S and at (or south of) 33°S, except when larval mortality is included. Nevertheless, compared to model configuration M0, adding growth in the dispersal model and a settlement criterion based on minimal length (model configuration M2) lead to an increase of the mean connectivity values (Table 2). However, when considering relative transport success (i.e., transport success relative to site with highest transport success for each model configuration), a decrease of the loco larval transport success is observed in the southern part of the domain for M2 compared to M0, particularly from the release areas located between 28°S to 38°S (Fig 5A and 5B). When limited available habitat for loco is considered (M3), there is a decrease in transport success in the northern (to 19°S) and southern (from 33°S) areas of the study domain (Fig 5C). Between 19°S and 22°S, connectivity values to and from this zone are high, *i*.*e*. there is a strong larval transport success from this zone and strong settlement to this zone (Fig 6C). Including fecundity ratio notably increases transport success for larvae released in southern areas, but does not remove the overall north-south gradient in transport success (M4; Fig 5D). When DVM behavior is integrated (M5; Figs 5E and 6E), transport success and connectivity values in the southern part of the study domain (from 28°S) decrease again compared to previous model configuration. The northward transport is marked all along the study domain (Fig 6E) and a very low transport success is obtained for larvae released around 29−30°S (Fig 5E). High connectivity values are still observed between 19°S and 22°S. Finally, when larval mortality is included (M6; Fig 6F), connectivity values of loco larvae dramatically decrease and are around 120,000 times smaller than values obtained without mortality. Qualitatively, there is a steady decrease in connectivity from 23°S to the southern limit of the domain, with very low levels of transport success observed south of 29°S (Fig 5F).

### Loco subpopulations

**Fig 7:**
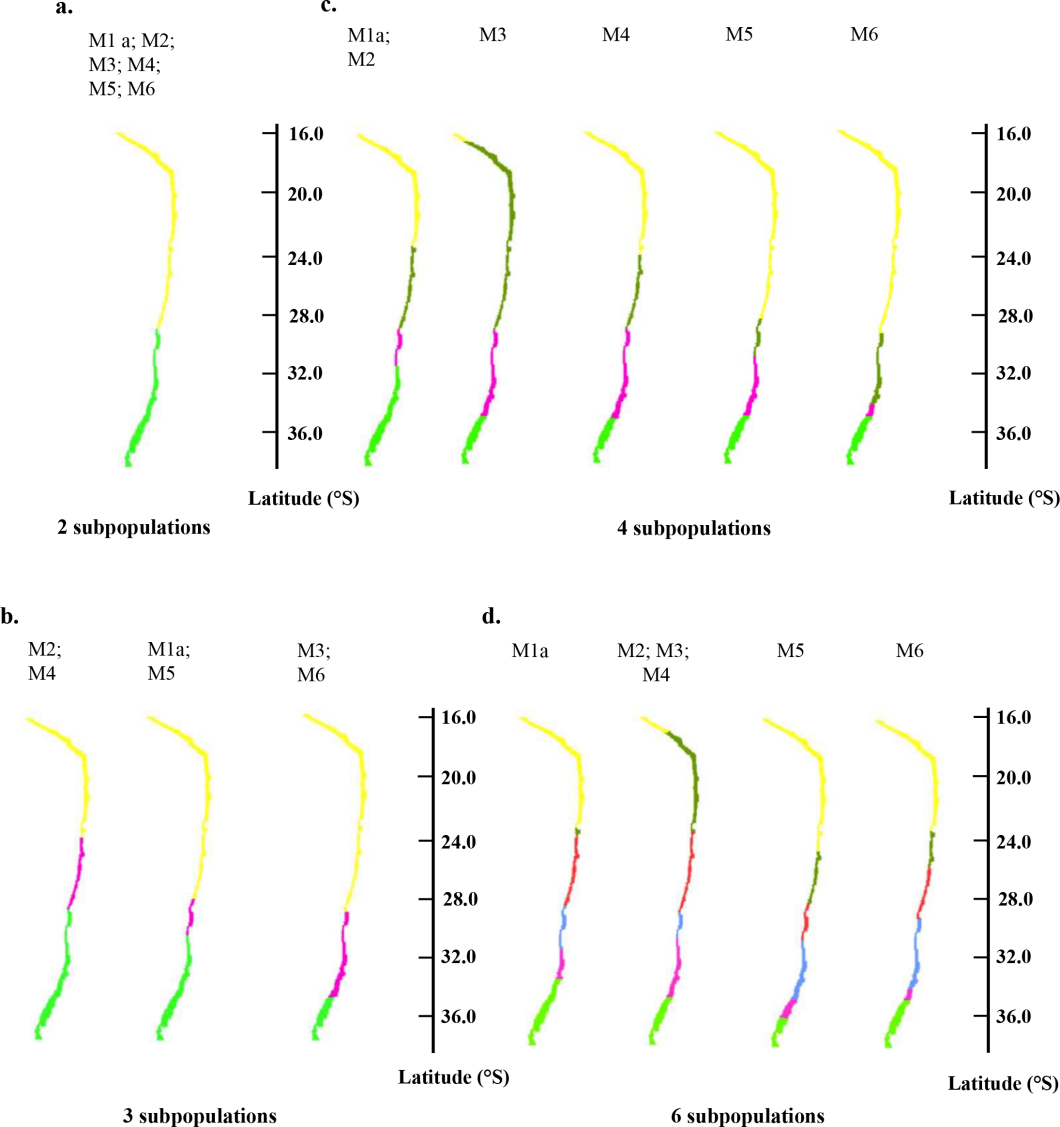
Partitioning of the loco population into 2 (a.), 3 (b.), 4 (c.), and 6 (d.) subpopulations successively identified from the connectivity matrices obtained with model configurations M1a (with DVM 0–20 m), M2 (with growth), M3 (adding habitat), M4 (adding fecundity), M5 (adding DVM), and M6 (adding mortality).

From the connectivity results obtained for each model configuration, we partitioned into 2 to 6 subpopulations the loco population along the Chilean coast (Fig 7). The first separation is obtained above or below the region around 29−30°S for all model configurations (Fig 7A). A separation is observed at ~17°S for M3 from 4 subpopulations (Fig 7C) and for M2 and M4 from 6 subpopulations (Fig 7D), *i*.*e*. when DVM and mortality are not considered. From 4 subpopulations (Fig 7C and 7D), a recurrent separation appears around 23°S (except for M5) and a separation is observed around 35°S (except for M1a). When DVM is included, the southern part of the domain (from 29°S) is divided into 3 subpopulations instead of 2 when it is not (Fig 7C and 7D).

## Discussion

Determining adequate spatial units for stock assessment and monitoring is a priority issue for science and managers worldwide [51–53] and particularly in Chile. Though biophysical dispersal models play an important role in making such determinations [4], realistic biophysical models including a suite of complex biological processes are relatively rare and have received little attention compared to purely-hydrodynamic, passive drift models. This missing complexity in passive drift models is one presumed impediment to their use in making management evaluations. The main objective of our study was to assess the influence of several biological characteristics potentially important for larval transport on simulated connectivity patterns for loco, a highly exploited benthic species in Chile. Our results show consistent relative spatial patterns of connectivity for all model configurations, with relatively well-defined areas of high connectivity and an overall decrease in transport success to settlement areas from North to South. Nevertheless, DVM and larval mortality significantly affected absolute connectivity levels accentuating the contrast between areas North and South of 30°S. Dispersal distances and connectivity patterns vary depending on the biological factors considered in our larval dispersal model, with a rigid surface/at-depth DVM scheme leading to significantly decreased transport success and increased dispersal distances. Recurrent loco subpopulations separations around 17°S, 23°S, 29−30°S, and 35°S suggest that these areas represent real dispersal barriers for loco.

Loco connectivity patterns are quantitatively dependent on biological factors implemented in the model and vary spatially from North to South. Among all the model configurations examined in this study, DVM was one of the biological processes that most heavily influenced loco larvae dispersal patterns. In both simulations M1 and M5, adding a DVM increased the mean dispersal distances (Table 2) compared to the corresponding model configurations without a DVM. This result is likely due to the presence of the equatorward near-surface Chile Coastal Current [54–55] with alongshore velocities being higher on average in the 0–20 m depth range. The two peaks of alongshore velocity observed at 23°S and 30°S are consistent with the two peaks in dispersal distances observed around these locations when DVM is included.

Adding a DVM also produced a reduction of the global transport success compared to the corresponding model configurations without a DVM. Increasing the DVM vertical range does increase transport success, but values do not exceed those for the model without DVM for the set of DVM vertical ranges assessed in this study. Our results on transport success are consistent with a modeling study from the upwelling region of California that also found that DVM behavior decreased recruitment [56]. Carr *et al*. [56] concluded that this was because increased nighttime dispersal due to exposure to the fast moving Ekman layer is not compensated by decreased transport velocities during the day while at depth. In contrast to our model with rigid surface DVM scheme, settlers are distributed deeper in the column water during transport in configurations without DVM. In the hydrodynamic model, we observed that the mean velocities of the cross-shore currents comprised between 20 and 60 m are weakly positive (onshore) resulting in higher larval settlement without DVM. In another study off Chile, higher settlement in simulations with DVM (from the surface to 50 m) was obtained than without [11]. These contrasting results could also be explained by the initial depth distribution used in this study [11] (surface release) and our (0 to 20 m depth release) simulations. Indeed, in our study, the larval settlement also significantly depends on the larval release depth with for example almost half the amount of larvae recruited for release depth 0–5m than for 15–20m. DVM leads to increased settlement when compared to simulations without DVM and release at the surface only [11], but to decreased settlement when compared to simulations without DVM and release over a range of surface and sub-surface depths, like here.

One striking aspect of our simulations is that adding biological processes such as growth and mortality produced a strong gradient of decreasing transport success as one moves from north to south in the study domain. Temperature gradients along the Chilean coast [29] produce predictable impacts on connectivity contrasts between North and South. Larvae in warmer northern waters have faster growth, and hence shorter dispersal times to settlement, leading to decreased larval exposure to offshore surface currents, ultimately producing lower mortality rates compared to those of larvae experiencing colder, southern waters. In the southern part of Chile (34–39°S), plankton was mainly observed in the offshore mesoscale oceanic features [57,58]. In the hydrodynamic model, offshore currents are dominant in surface waters where offshore transport is stronger and distributed over a deeper layer in the southern part than in the northern part of the domain in relation with a stronger offshore wind (Fig C in S1 File). Around 30°S (Coquimbo bay), a zone associated with intense upwelling, connectivity values are very low, particularly when DVM is implemented. However, this region has been described as a zone of intense recruitment for loco [59]. In our hydrodynamic model, wind stress forcing is from satellite measurements and therefore its very nearshore spatial structure is certainly inaccurate [60] (S2 File). The nearshore wind extrapolation results in an overestimated coastal wind stress and consequently a possibly too deep mixed layer (S2 File). In our model solutions, the mixed layer depth is deeper than 20 m, as observed in S2 File. With a DVM scheme between 0 and 20 m, larvae are likely to stay in the mixed layer *i*.*e*. they are mostly transported offshore. With a deeper DVM scheme as tested in model configurations M1b and M1c, our results showed higher connectivity values around this zone. Smaller-scale hydrodynamic simulations using fine-scale winds may be needed in this area to accurately determine transport dynamics.

Including relative fecundity (M4) leads to a slight relative increase in transport success south of 30°S due to higher reproductive outputs in this region [34], but does not eliminate the overall north-south decreasing tendency in transport success. Nevertheless, the effect of fecundity on connectivity from north to south is of relatively similar magnitude to that of temperature-dependent growth, and, therefore, our results are consistent with the hypothesis that relative fecundity plays a role in compensating for increased larval loss and mortality to the south.

The consistency of seasonality results across model configurations (Fig A in S1 File for DVM model configurations, similar results were obtained for other configurations; see also Garavelli *et al*. [33]) indicates that seasonality in connectivity for loco is mainly affected by hydrodynamic factors, not larval behavior. The annual larval release cycle of loco (March to August) is in phase with that of increased simulated retention. Recruited larvae disperse from austral autumn to spring, a period of downwelling generated by northerly winds off Chile [61,48]. Reproductive cycles are usually tuned to environmental cycles that are most determinant for successful recruitment [62,63], operating at either adult or larval stages [64,65]. For larval recruitment success, several environmental factors besides transport are usually considered, including food availability, temperature, and presence of predators [6]. However, transport is considered to be one of the most important environmental factors modulating larval success variability in upwelling areas [66,11]. The coincidence of the seasonality of observed loco reproduction and simulated retention patterns in surface and sub-surface waters is consistent with physical transport being a major determining factor for overall loco settlement success.

The identification of loco subpopulations from the connectivity matrices highlighted several robust patterns in the partitioning along the coast, which are also consistent with those obtained by Garavelli *et al*. [33]. In all simulations, a common major separation between subpopulations was obtained around 29−30°S. A second robust separation was observed around 23°S. These two regions are associated to a particular coastal topography with the presence of a bay at 30°S (Coquimbo bay) and a cape at 23°S (Mejillones peninsula). They are also characterized by an offshore Ekman transport inducing upwelling and impacting larval dispersal [54,67]. The combined effect of the latitudinal mesoscale variations of the upwelling favorable wind (i.e. offshore Ekman transport) and the coastal topography (i.e. the settlement area width) can strongly constrain the recruitment and so can explain several of its marked spatial patterns (Fig D in S1 File) and separations between subpopulations [68]. The other separations observed in some model configurations can be explained by low connectivity values obtained in the connectivity matrices due to low available habitat in these model configurations (particularly for M3 and M4; north of 17°S and south of 35°S). Globally, geomorphological features along the Chilean coast explain the robust separations observed in all cases. Biological processes, such as DVM and mortality, split the southern part of the domain in more subpopulations likely due to low connectivity values in this region, but did not affect major separations linked to geomorphological features.

Evaluating the spatial structure, dynamics and connectivity for populations with wide and continuous distribution ranges presents an important practical problem in biophysical modeling: a large domain is required to evaluate large scale patterns and identify meaningful subpopulation units defined by strong internal demographic connectivity. Nevertheless, model resolution for large domains is still constrained due to computing technology, and low-resolution hydrodynamic configurations have difficulty capturing some relevant coastal processes for retention and connectivity [69–71]. Therefore, broadscale connectivity patterns as those presented in this study should orient future biophysical efforts, pointing the relevant zones where higher resolution, nested hydrodynamic configurations should be implemented. Using a higher-resolution hydrodynamic model (S2 File; [33]) would be an important improvement that may help understand why the central part of Chile, around 30°S, has low larval settlement in our model. The spatial structure of loco population depends on both pre- and post-recruitment processes so local adult density and abundance do not necessarily reflect recruitment levels. Nevertheless, given the heavy exploitation of loco throughout most of our study domain, it is expected that sites of high abundance sustaining important landings also present high recruitment (while the contrary is not necessarily true). Also, in our hydrodynamic model, interannual forcing (e.g. ENSO-related) is absent. Gaymer *et al*. [72] observed low recruitment levels of several benthic species in northern Chile during La Niña, likely caused by increased upwelling intensity leading to offshore larval losses. Including such variability in our model could modify the observed patterns of loco connectivity. Another limit of our model is the use of larval mortality dependent on growth that is driven by seawater temperature only. Larval mortality is also known to be highly dependent on food availability, which may be included in our model by coupling it with estimates of primary and secondary productivity and implementing a bioenergetic model of larval growth and mortality [73]. However, globally, biological factors successively included in our larval dispersal model to describe loco connectivity allow representing general patterns of loco distribution along the Chilean coast. Loco connectivity is significantly more important in the northern part than in the southern part of Chile due to the combination of currents direction, seawater temperatures and high levels of available habitat. The general tendency of contrasting connectivity between northern and southern parts of Chile and the description of different subpopulations suggest that management should be appropriately adapted for each of these zones.

## Supporting Information

S1 File Contains Figs A, B, C, and D. Transport success of loco larvae in relation to area and month of release for model configuration M1a (Fig A). Distribution of length at settlement for loco larvae after 140 days of planktonic larval duration for model configuration M2 (Fig B). Vertical sections of the annual mean of the cross-shore component of current velocity (m.s^-1^) averaged between 16°S and 29°S (a.) and 30°S and 38°S (b.) for the hydrodynamic model used. Positive values indicate onshore transport. Black contours represent onshore speed = 0 m.s^-1^. Gray contours indicate temperature isotherms. (Fig C). Ratio between the Ekman velocity U_ek_ (m.s^-1^) and the settlement area width Ls (m) as a function of latitude. U_ek_ is estimated by τ / (ρ f h_bl_) and averaged over the settlement area, with τ the wind stress, ρ the density and h_bl_ the depth of the mixed-layer. This ratio is an indicator of the combined effect of the upwelling favorable wind and the coastal topography on the near-surface transport out of the settlement area (Fig D).
(DOCX)

S2 File We described a high-resolution hydrodynamic model off Central Chile (30°S). We represented the annual mean of wind stress magnitude from satellite data and from high resolution model between 29°S and 31°S. Also, the mixed depth layer from high resolution ROMS model forced by satellite wind data and forced by high-resolution WRF atmospheric model wind are described.
(DOCX)

## References

[1] Hastings A, Botsford LW. Persistence of spatial populations depends on returning home. Proc Natl Acad Sci USA. 2006;103: 6067–6072. 16608913 10.1073/pnas.0506651103 PMC1458697

[2] Pineda J, Hare J, Sponaugle S. Larval Transport and Dispersal in the Coastal Ocean and Consequences for Population Connectivity. Oceanography. 2007;20: 22–39.

[3] Siegel D, Kinlan B, Gaylord B, Gaines S. Lagrangian descriptions of marine larval dispersion. Mar Ecol Prog Ser. 2003;260: 83–96.

[4] Cowen RK, Paris CB, Srinivasan A. Scaling of connectivity in Marine Populations. Science. 2006;311: 522–27. 16357224 10.1126/science.1122039

[5] Aiken CM, Navarrete S, Castillo M, Castilla JC. Along-shore larval dispersal kernels in a numerical ocean model of the central Chilean coast. Mar Ecol Prog Ser. 2007;339: 13–24.

[6] Metaxas A, Saunders M. Quantifying the "bio-" components in biophysical models of larval transport in marine benthic invertebrates: advances and pitfalls. The Biological Bulletin. 2009;216: 257–272. 19556593 10.1086/BBLv216n3p257

[7] Botsford LW, Castilla JC, Peterson CH. The Management of Fisheries and Ecosystems. Science. 1997;277: 509–515.

[8] Castilla JC. Coastal marine communities: trends and perspectives from human-exclusion experiments. Trends Ecol Evol. 1999;14: 280–283. 10370266 10.1016/s0169-5347(99)01602-x

[9] Peliz A, Marchesiello P, Dubert J, Marta-Almeida M, Roy C, Queiroga H. A study of crab larvae dispersal on the Western Iberian Shelf: Physical processes. J Mar Syst. 2007;68: 215–236.

[10] Butler MJ IV, Paris C, Goldstein J, Matsuda H, Cowen RK. Behavior constrains the dispersal of long-lived spiny lobster larvae. Mar Ecol Prog Ser. 2011;422: 223–237.

[11] Aiken CM, Navarrete SA, Pelegrí JL. Potential changes in larval dispersal and alongshore connectivity on the central Chilean coast due to an altered wind climate. J Geophys Res. 2011;116: 1–14, 10.1029/2011JG001731

[12] Robins PE, Neill SP, Gime L, Jenkins SR, Malham SK. Physical and biological controls on larval dispersal and connectivity in a highly energetic shelf sea. Limnol Oceanogr. 2013;58: 1–21.

[13] Parada C, Mullon C, Roy C, Fréon P, Hutchings L, van der Lingen CD. Does vertical migratory behaviour retain fish larvae onshore in upwelling ecosystems? A modeling study of anchovy in the southern Benguela. Afr. J. Mar. Sci. 2008;30: 437–452.

[14] Yannicelli B, Castro L, Parada C, Schneider W, Colas F, Donoso D. Distribution of *Pleuroncodes monodon* larvae over the continental shelf of south-central Chile: Field and modeling evidence for partial local retention and transport. Prog Oceanogr. 2012;92–95: 206–227.

[15] Rumrill SS. Natural mortality of marine invertebrate larvae. Ophelia. 1990;32: 163–198.

[16] Pedersen TM, Hansen JLS, Josefson AB, Hansen BW. Mortality through ontogeny of soft-bottom marine invertebrates with planktonic larvae. J Mar Syst. 2008;73: 185–207.

[17] Guizien K, Brochier T, Duchêne J, Koh B, Marsaleix P. Dispersal of *Owenia fusiformis* larvae by wind-driven currents: turbulence, swimming behaviour and mortality in a three-dimensional stochastic model. Mar Ecol Prog Ser. 2006;311: 47–66.

[18] Ellien C, Thiébaut E, Dumas F, Salomon JC, Nival P. A modelling study of the respective role of hydrodynamic processes and larval mortality on larval dispersal and recruitment of benthic invertebrates: example of *Pectinaria koreni* (Annelida: Polychaeta) in the Bay of Seine (English Channel). J Plankton Res. 2004;26: 117–132.

[19] Pechenik JA, Lima GM. Relationship between growth, differentiation, and length of larval life for individually reared larvae of the marine gastropod, *Crepidula fornicata*. The Biological Buletin. 1984;166: 537–549.

[20] O’Connor MI, Bruno JF, Gaines SD, Halpern BS, Lester SE, Kinlan BP, Weiss JM. Temperature control of larval dispersal and the implications for marine ecology, evolution, and conservation. Proc Natl Acad Sci USA. 2007;104: 1266–1271. 17213327 10.1073/pnas.0603422104 PMC1764863

[21] Incze L, Naimie E. Modelling the transport of lobster (*Homarus americanus*) larvae and postlarvae in the Gulf of Maine. Fish Oceanogr. 2000;9: 99–113.

[22] Domingues CP, Nolasco R, Dubert J, Queiroga H. Model-derived dispersal pathways from multiple source populations explain variability of invertebrate larval supply. Plos One. 2012;7: e35794 10.1371/journal.pone.0035794 22558225 PMC3338459

[23] Nolasco R, Dubert J, Domingues C, Cordeiro Pires A, Queiroga H. Model-derived connectivity patterns along the western Iberian Peninsula: asymmetrical larval flow and source-sink cell. Mar Ecol Prog Ser. 2013;485: 123–142.

[24] Leiva G, Castilla JC. A review of the world marine gastropod fishery: evolution of catches. Rev. Fish Biol. Fish. 2002;11: 283–300.

[25] Castilla JC, Fernández M. Small-scale benthic fisheries in Chile: on co-management and sustainable use of benthic invertebrates. Ecol Appl. 1998;8: 124–132.

[26] Gallardo Fernández G.L. From Seascapes of Extinction to Seascapes of Confidence Territorial Use Rights in Fisheries in Chile: El Quisco and Puerto Oscuro. Co-Action Publishing. Aberystwyth, Wales: Cambrian Printers Ltd; 2008.

[27] Molinet C, Niklitschek E, Moreno CA, Arévalo A. Vertical distribution of early and competent larvae of *Concholepas concholepas* in two systems of Chilean inland seas. Marine Biology. 2008;153: 779–787.

[28] Manríquez PH, Castilla JC. Behavioural traits of competent *Concholepas concholepas* (loco) larvae. Mar Ecol Prog Ser. 2011;430: 207–221.

[29] Fernández M, Jaramillo E, Marquet PA, Moreno CA, Navarrete SA, Ojeda FP, Valdovinos CR, Vasquez JA. Diversity, dynamics and biogeography of Chilean benthic nearshore ecosystems: an overview and guidelines for conservation. Rev. Chil. Hist. Nat. 2000;73: 797–830.

[30] DiSalvo LH. Observations on the larval and post-metamorphic life of *Concholepas concholepas* (Bruguière, 1789). Veliger. 1988;30: 358–368.

[31] Moreno CA, Asencio G, Ibañez S. Patrones de asentamiento de *Concholepas concholepas* (Brugière) (Mollusca: Muricidae) en la zona intermareal rocosa de Valdivia, Chile. Rev. Chil. Hist. Nat. 1993;66: 93–101.

[32] Molinet C, Arévalo A, González MT, Moreno CA, Arata J, Niklitschek E. Patterns of larval distribution and settlement of *Concholepas concholepas* (Bruguiere, 1789) (Gastropoda, Muricidae) in fjords and channels of southern Chile. Rev. Chil. Hist. Nat. 2005;78: 409–423.

[33] Garavelli L, Kaplan DM, Colas F, Stotz W, Yannicelli B, Lett C. Identifying appropriate spatial scales for marine conservation and management using a larval dispersal model: the case of *Concholepas concholepas* (loco) in Chile. Prog Oceanogr. 2014;124: 42–53. 10.1016/j.pocean.2014.03.011

[34] Fernández M, Calderón R, Cancino J, Jeno K. The effect of temperature on the development of encapsulated embryos of *Concholepas concholepas* along a latitudinal cline. Mar Ecol Prog Ser. 2007;348: 229–237.

[35] Shchepetkin AF, McWilliams JC. The Regional Oceanic Modeling System: a split-explicit, free-surface, topography-following-coordinate ocean model. Ocean Modelling. 2005;9: 347–404. 10.1016/j.ocemod.2004.08.002

[36] Shchepetkin AF, McWilliams JC. Correction and commentary for ‘‘Ocean forecasting in terrain-following coordinates: formulation and skill assessment of the regional ocean modeling system” by Haidvogel et al J Comp Phys 227:3595–3624. J Comput Phys. 2009;228: 8985–9000 10.1016/j.jcp.2009.09.002

[37] Marchesiello P, McWilliams JC, Shchepetkin AF. Equilibrium structure and dynamics of the California Current System. J Phys Oceanogr. 2003;33: 753–783.

[38] Penven P, Echevin V, Pasapera J, Colas F, Tam J. Average circulation, seasonal cycle, and mesoscale dynamics of the Peru Current System: a modeling approach. J Geophys Res. 2005;110 10.1029/2005JC002945110

[39] Mason E, Molemaker MJ, Shchepetkin AF, Colas AF, McWilliams JC, Sangra P. Procedures for offline grid nesting in regional ocean models. Ocean Modelling. 2010;35: 1–15. doi: 10.1016/ j.ocemod.2010.05.007

[40] Veitch J, Penven P, Shillington F. Modelling equilibrium dynamics of the Benguela Current System. J Phys Oceanogr. 2010;40: 1942–1964. 10.1175/2010JPO4382.1

[41] Da Silva AM, Young CC, Levitus S. Atlas of Surface Marine Data 1994, vol. 1. Algorithms and Procedures, Technical Report. National Oceanographic and Atmospheric Administration. Silver, Spring, MD; 1994.

[42] Risien CM, Chelton DB. A global climatology of surface wind and wind stress fields from eight years of QuikSCAT scatterometer data. J Phys Oceanogr. 2008;38: 2379–2413. doi: 10.1175/2008. JPO3881.1

[43] Carton J, Giese B. A reanalysis of ocean climate using Simple Ocean Data Assimilation (SODA). Monthly Weather Review. 2008;136: 2999–3017. 10.1175/2007MWR1978.1

[44] Colas F, McWilliams JC, Capet X, Kurian J. Heat Balance and eddies in the Peru-Chile current system. Clim Dyn. 2012;

[45] Colas F, Capet X, McWilliams JC, Li Z. Mesoscale eddy buoyancy flux and eddy-induced circulation in Eastern Boundary Currents. J Phys Oceanogr. 2013;43: 1073–1095, doi: 10.1175/JPO-D-11-0241.1.

[46] Lett C, Verley P, Mullon C, Parada C, Brochier T, Penven P, Blanke B. A Lagrangian tool for modelling ichthyoplankton dynamics. Environ Model Softw. 2008;23: 1210–1214.

[47] DiSalvo LH, Carriker MR. Planktonic, metamorphic, and early benthic behavior of the Chilean loco *Concholepas concholepas* (Muricidae, Gastropoda, Mollusca). J. Shellfish Res. 1994;13: 57–66.

[48] Thiel M, Macaya EC, Acuna E, Arntz WE, Bastias H, Brokordt K, et al The Humboldt Current system of northern and central Chile. Oceanographic processes, ecological interactions and socioeconomic feedback. Oceanogr Mar Biol Annu Rev. 2007;45: 195–344.

[49] Manríquez PH, Navarrete SA, Rosson A, Castilla JC. Settlement of the gastropod *Concholepas concholepas* on shells of conspecific adults. J Mar Biol Assoc U.K. 2004;84: 651–658.

[50] Jacobi MN, André C, Döös K, Jonsson PR. Identification of subpopulations from connectivity matrices. Ecography. 2012;35: 1004–1016.

[51] Green A, Smith SE, Lipsett-Moore G, Groves C, Peterson N, Sheppard S, et al Designing a resilient network of marine protected areas for Kimbe Bay, Papua New Guinea. Oryx. 2009;43: 488–498.

[52] Ban NC, Adams VM, Alamany GR, Ban S, Cinner JE, McCook LJ, et al Designing, implementing and managing marine protected areas: Emerging trends and opportunities for coral reef nations. J Exp Mar Bio Ecol. 2011;408: 21–31.

[53] White C, Costello C. Matching spatial property rights fisheries with scales of fish dispersal. Ecol Appl. 2011;21: 350–362. 21563568 10.1890/09-1188.1

[54] Strub PT, Mesias JM, Montecino V, Ruttlant J, Salinas S. Coastal ocean circulation of western south America, in The Sea, vol. 11, The Global Coastal Ocean: Regional Studies and Syntheses, edited by Robinson A.R. and Brink K.H.. John Wiley, Hoboken, N.J; 1998 pp. 273–313.

[55] Shaffer G, Hormazabal S, Pizarro O, Salinas S. Seasonnal and interannual variability of currents and temperature off central Chile. J Geophys Res. 1999;104: 951–961.

[56] Carr S, Capet X, McWilliams JC, Pennington JT, Chavez FP. The influence of diel vertical migration on zooplankton transport and recruitment in an upwelling region: estimates from a coupled behavioral-physical model. Fish Oceanogr. 2008;17: 1–15.

[57] Morales CE, González HE, Hormazabal SE, Yuras G, Letelier J, Castro LR. The distribution of chlorophyll-a in the coastal transition zone off Concepción, central Chile, during different oceanographic conditions. Prog Oceanogr. 2007;75: 452–469.

[58] Morales CE, Torreblanca ML, Hormazabal S, Correa-Ramírez M, Nuñez S, Hidalgo P. Mesoscale structure of copepod assemblages in the coastal transition zone and oceanic waters off central-southern Chile. Prog Oceanogr. 2010;84: 158–173.

[59] González J, et al.; 2005 Informe Proyecto FIP 2002–16 Bases biológicas para la evaluación y manejo de metapoblaciones de loco en la III y IV Regiones. Instituto de Fomento Pesquero.

[60] Capet X, Marchesiello P, McWilliams JC. Upwelling response to coastal wind profiles. Geophys Res Lett. 2004;31 10.1029/2004GL020123

[61] Castro LR, Salinas G, Hernández H. Environmental influences on winter spawning of the anchoveta *Engraulis ringens* off central Chile. Mar Ecol Prog Ser. 2000;197: 247–258.

[62] Cushing DH. Plankton production and year-class strength in fish populations: an update of the match/mismatch hypothesis. Adv Mar Biol. 1990;9: 295–354.

[63] Bakun A. Patterns in the ocean: ocean processes and marine population dynamics. University of California Sea Grant Program, San Diego, California, USA, in cooperation with Centro de Investigaciones Biologicas de Noroeste, La Paz, Baja California Sur, Mexico; 1996.

[64] Sakurai Y, Kiyofuji H, Saitoh S, Goto T, Hiyama Y. Changes in inferred spawning areas of *Todarodes pacificus* (Cephalopoda: Ommastrephidae) due to changing environmental conditions. ICES J Mar Sci. 2000;57: 24–30.

[65] Brochier T, Colas F, Lett C, Echevin V, Cubillos LA, Tam J, et al Small pelagic fish reproductive strategies in upwelling systems: A natal homing evolutionary model to study environmental constraints. Prog Oceanogr. 2009;83: 261–269.

[66] Brochier T, Lett C, Tam J, Fréon P, Colas F, Ayón P An individual-based model study of anchovy early life history in the northern Humboldt Current system. Prog Oceanogr. 2008;79: 313–325.

[67] Letelier J, Pizarro O, Nuñez S. Seasonal variability of coastal upwelling and the upwelling front off central Chile. J Geophys Res. 2009;114: C12009 10.1029/2008JC005171

[68] Navarrete SA, Wieters EA, Broitman BR, Castilla JC. Scales of benthic-pelagic coupling and the intensity of species interactions: From recruitment limitation to top-down control. Proc Natl Acad Sci USA. 2005;102: 046–051. 10.1073/pnas.0509119102 PMC1312419 16332959

[69] Kaplan DM, Largier JL, Navarrete S, Guiñez R, Castilla JC. Large diurnal temperature fluctuations in the nearshore water column. Estuar Coast Shelf Sci. 2003;57: 385–398.

[70] Rutllant JA, Rosenbluth B, Hormazabal S. Intraseasonal variability of wind-forced coastal upwelling off central Chile (30°S). Cont Shelf Res. 2004;24: 789–804.

[71] Nickols KJ, White JW, Largier JL, Gaylord B. Marine Population Connectivity: Reconciling Large-Scale Dispersal and High Self-Retention. Am Nat. 2015;185: 196–211. 10.1086/679503 25616139

[72] Gaymer CF, Palma AT, Vega JMA, Monaco CJ, Henríquez LA. Effects of La Nina on recruitment and abundance of juveniles and adults of benthic community-structuring species in northen Chile. Mar. Freshw. Res. 2010;61: 1185–1196.

[73] Kooijman SALM Dynamic Energy Budget Theory for Metabolic Organisation. Third Edition Cambridge University Press 514 pp; 2010.

