# Supplementary material for: Influence of Biological Factors on Connectivity Patterns for Concholepas concholepas (loco) in Chile
Source: PLoS One. 2016 Jan 11;11(1):e0146418. doi: 10.1371/journal.pone.0146418 (PMC4713471; doi:10.1371/journal.pone.0146418)
Supplement: S1 File — Transport success of loco larvae in relation to area and month of release for model configuration M1a (Fig A). Distribution of length at settlement for loco larvae after 140 days of planktonic larval duration for model configuration M2 (Fig B). Vertical sections of the annual mean of the cross-shore component of current velocity (m.s-1) averaged between 16°S and 29°S (a.) and 30°S and 38°S (b.) for the hydrodynamic model used. Positive values indicate onshore transport. Black contours represent onshore speed = 0 m.s-1. Gray contours indicate temperature isotherms. (Fig C). Ratio between the Ekman velocity Uek (m.s-1) and the settlement area width Ls (m) as a function of latitude. Uek is estimated by τ / (ρ f hbl) and averaged over the settlement area, with τ the wind stress, ρ the density and hbl the depth of the mixed-layer. This ratio is an indicator of the combined effect of the upwelling favorable wind and the coastal topography on the near-surface transport out of the settlement area (Fig D). (DOCX) [file pone.0146418.s001.docx]

# Influence of biological factors on connectivity patterns for *Concholepas concholepas* (loco) in Chile

Lysel Garavelli, François Colas, Philippe Verley, David Michael Kaplan, Beatriz Yannicelli, Christophe Lett

**S1 File**

Figure A: Transport success of loco larvae in relation to area and month of release for model configuration M1a.

Figure B: Distribution of length at settlement for loco larvae after 140 days of planktonic larval duration for model configuration M2

Figure C: Vertical sections of the annual mean of the cross-shore component of current velocity (m.s^-1^) averaged between 16°S and 29°S (a.) and 30°S and 38°S (b.) for the hydrodynamic model used. Positive values indicate onshore transport. Black contours represent onshore speed = 0 m.s^-1^. Gray contours indicate temperature isotherms.

Figure D: Ratio between the Ekman velocity U_ek_ (m.s^-1^) and the settlement area width Ls (m) as a function of latitude. U_ek_ is estimated by τ / (ρ f h_bl_) and averaged over the settlement area, with τ the wind stress, ρ the density and h_bl_ the depth of the mixed-layer. This ratio is an indicator of the combined effect of the upwelling favorable wind and the coastal topography on the near-surface transport out of the settlement area.
