# Supplementary material for: Influence of Biological Factors on Connectivity Patterns for Concholepas concholepas (loco) in Chile
Source: PLoS One. 2016 Jan 11;11(1):e0146418. doi: 10.1371/journal.pone.0146418 (PMC4713471; doi:10.1371/journal.pone.0146418)
Supplement: S2 File — We represented the annual mean of wind stress magnitude from satellite data and from high resolution model between 29°S and 31°S. Also, the mixed depth layer from high resolution ROMS model forced by satellite wind data and forced by high-resolution WRF atmospheric model wind are described. (DOCX) [file pone.0146418.s002.docx]

# Influence of biological factors on connectivity patterns for *Concholepas concholepas* (loco) in Chile

Lysel Garavelli, François Colas, Philippe Verley, David Michael Kaplan, Beatriz Yannicelli, Christophe Lett

**S2 File.**

To illustrate the sensitivity of the mixed-layer depth to the wind structure in the coastal region off Central Chile (30°S) we present here results from a high-resolution hydrodynamic model forced by two different wind stress products. The high-resolution hydrodynamic model solutions are obtained with the ROMS model following an offline nesting approach (Mason et al., 2010): a small coastal domain (2° x 2°), with 1 km horizontal resolution, centered around 30°S is embedded in a larger domain with 3 km horizontal resolution, itself embedded in the large domain used in the present study (at 7.5 km resolution). A description of the 3 km domain is given in Garavelli et al. [31]. Two different solutions on this 1 km domain, differing only in their wind stress forcing, are run for 3 years. The first solution (see Figure in Appendix S2 left column) is forced by the same wind stress than the ROMS parent solutions (the SCOW scatterometer-based climatology) interpolated on the 1 km grid. The second solution (right column) is forced by a monthly-mean wind stress climatology obtained from a high-resolution (4 km) regional atmospheric model. A suite of three embedded domains is configured with the WRF model (Skamarock et al., 2008) at 36, 12 and 4 km horizontal resolution (O. Astudillo, personal communication). The 4 km, smaller domain, is centered on the coastal region off Chile at 30°S. The model is run for 6 years (2007-2012) and the outputs are used to compute a monthly-mean wind stress climatology. This climatology is then interpolated on the 1 km ocean model grid. We do not give more details on the regional atmospheric model configuration because our purpose here is process-oriented and aims at illustrating the sensitivity of the mixed-layer response to two very contrasted near-shore wind structure. The scatterometer coastal wind structure is unrealistic as it results mainly from an extrapolation toward the coast of the offshore wind. It barely exhibits a near-shore reduction. On the other hand, the atmospheric model wind has a very pronounced near-shore reduction that may be overestimated but has the advantage to differ largely from the other forcing. Consequently, the near-shore mixed-layer is much deeper (20 m or more) when the model is forced with the scatterometer wind than in the other case (less than 10 m). In a coastal upwelling region, the mixed-layer turbulence is strongly wind-driven so the mixed layer depth can be used as a good proxy for the Ekman layer depth. This means that with a DVM scheme between 0 and 20 m, larvae are more prone to stay permanently in the Ekman layer depth and, thus, be transported offshore in the model solution forced with satellite wind. By contrast, in the hydrodynamic model solution forced by a reduced near-shore wind, the Ekman layer is much shallower in the coastal region so larvae with a DVM scheme between 0 and 20 m will be less subject to offshore transport.


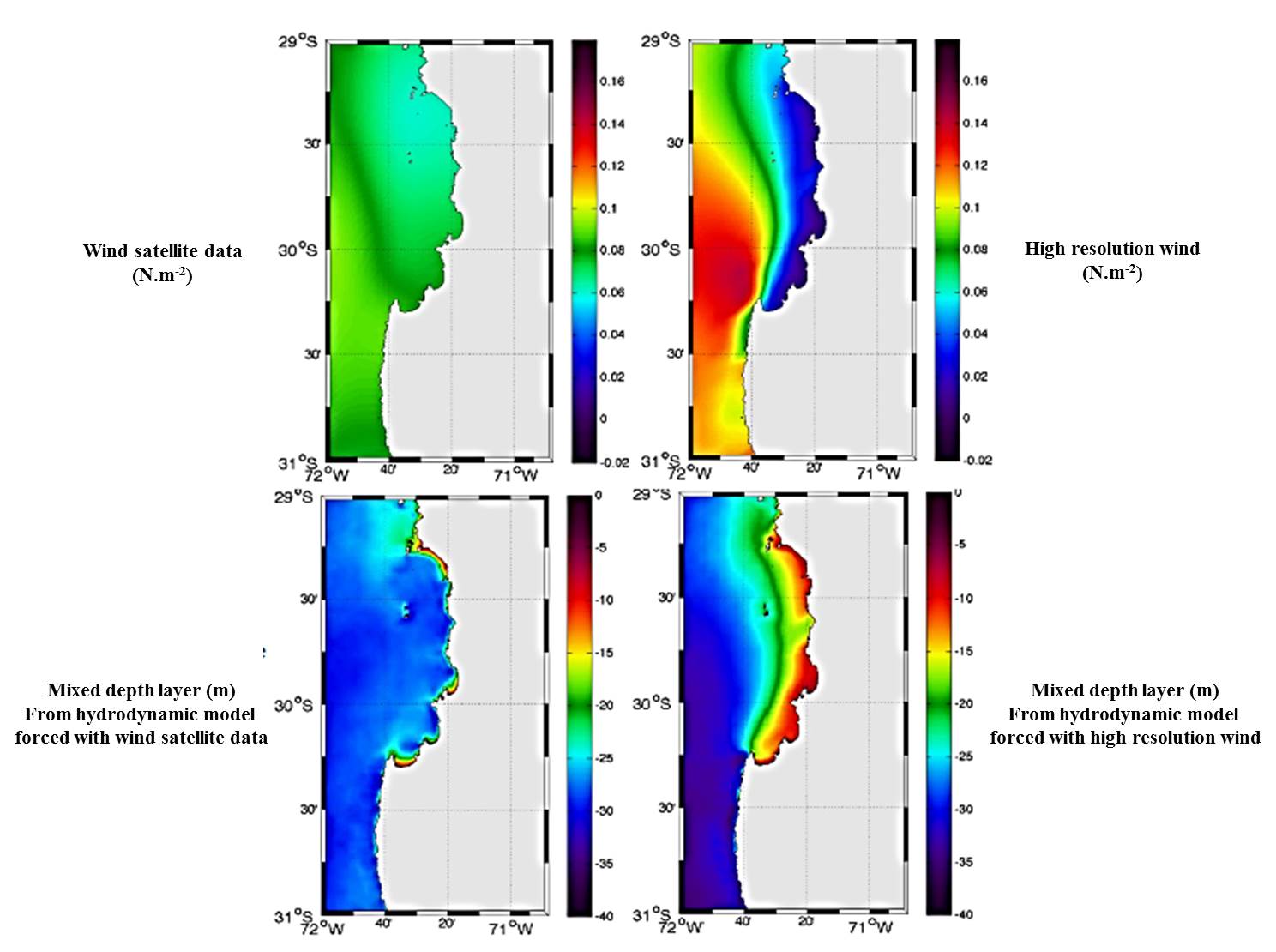


Figure A: Annual mean of wind stress magnitude (N.m^-^²) from satellite data (top left) and from high resolution model (top right) between 29°S and 31°S. Mixed depth layer (m) from high resolution ROMS model forced by satellite wind data (bottom left) and forced by high-resolution WRF atmospheric model wind (bottom right). Spatial resolution of high resolution WRF model wind and hydrodynamic ROMS model are 4 km and 1 km respectively.

References

Mason, E., Molemaker, M.J., Shchepetkin, A.F., Colas, A.F., McWilliams, J.C., Sangra, P. 2010. Procedures for ofﬂine grid nesting in regional ocean models. Ocean Modelling, 35: 1-15. doi:10.1016/ j.ocemod.2010.05.007.

Skamarock, W.C., *et al.* 2008. A description of the advanced research WRF version 3. Tech. Rep. NCAR/TN-475+STR. 113 pp.
